# Use of the forgotten joint score (FJS)-12 to evaluate knee awareness after quadriceps tendon reconstruction

**DOI:** 10.1186/s12891-023-06574-9

**Published:** 2023-05-30

**Authors:** Manuel P. Sava, Benjamin L. Schelker, Zainab A. Khan, Felix Amsler, Michael T. Hirschmann

**Affiliations:** 1 Department of Orthopedic Surgery and Traumatology, Kantonsspital Baselland, CH-4101 Bruderholz, Switzerland; 2 Department of Clinical Research, Research Group Michael T. Hirschmann, Regenerative Medicine & Biomechanics, University of Basel, CH-4001 Basel, Switzerland; 3 Research and Development Department, AO Hospital, Karachi, Pakistan; 4 Amsler Consulting, Gundeldingerrain 111, CH-4059 Basel, Switzerland

**Keywords:** Quadriceps Tendon rupture, Knee, Quadriceps tendon reconstruction, Forgotten Joint Score, Validation, WOMAC, Tegner, Lysholm, PROMs, Functional outcomes

## Abstract

**Background:**

Quadriceps tendon rupture (QTR) is a severe injury of the knee extensor apparatus. The study aims to validate the use of forgotten joint score (FJS-12) for functional outcome assessing after surgical treatment of QTR.

**Methods:**

Fifty-seven patients who underwent surgery for QTR with transosseous suture reconstruction in a single orthopaedic surgery and traumatology center between 2015 and 2020 were eligible for enrolment in this retrospective case series. The demographic data and other pre-operative details such as age, gender, comorbidities and medication use also were extracted from the medical records. Patient reported outcome measures (PROMs) were gathered in the form of Western Ontario and McMaster Universities Arthritis Index Score (WOMAC), Tegner Activity Score (TAS), Lysholm Score and FJS-12 at a mean follow-up time of 49.84 months ± 20.64 months. The FJS-12 was validated by correlation with WOMAC, TAS and Lysholm Score.

**Results:**

The mean age of all patients were 69.2 ± 13.6 years with 51 (89.5%) males and 6 (10.5%) females. The mean time from injury to surgery was 3.39 ± 5.46 days. All patients reported satisfactory functional outcomes after surgery on FJS-12, WOMAC and Lysholm scores, except the TAS, which decreased slightly from pre-operative level. There was a high negative correlation between WOMAC and FJS-12, but moderate positive correlations between FJS-12 and TAS and Lysholm scores. The Cronbach’s alpha value was 0.96 for 12 items in FJS-12.

**Conclusion:**

This study has found that FJS-12 is a reliable and easy to assess tool for functional outcomes after QTR reconstruction. It has shown moderate to strong correlation with other commonly used outcome measures (WOMAC, TAS and Lysholm).

## Background

Quadriceps tendon ruptures (QTR) are rather common injuries of the knee extensor apparatus [1]. The injury most frequently affects men over the age of 40 years [2]. QTR rarely occurs as a result of a directly exerted force to the tendon in the context of severe accidents [3]. The majority of the ruptures are the result of an indirect mechanism through a sudden and strong contraction of the quadriceps muscle during a jump, or via compensatory movements after a fall [4, 5]. However, the force exercised by the muscles alone is usually not strong enough to tear a healthy tendon. Pre-existing degenerative changes in the tendon though can weaken the tissue to such an extent that it can suffer tears [6, 7]. With mechanical overload, especially in jumping sports (e.g. basketball and high jump), QTR can also occur at a younger age due to repetitive micro trauma to the quadriceps tendon (QT) [8, 9]. Complete QTR requires timely surgical treatment, as delays have been shown to be associated with worse outcomes [10, 12]. For partial ruptures, conservative treatment can be considered [9]. Nonetheless, it has been reported that in the case of partial ruptures, debridement and suturing of the rupture can lead to an improved outcome [11, 13].

There are different patient reported outcomes measures (PROMs) used for assessment of functional outcomes after knee surgeries, such as Western Ontario and McMaster Universities Osteoarthritis Index (WOMAC), Tegner, Lysholm, Knee Injury and Osteoarthritis Outcome Score (KOOS), Forgotten Joint Score- 12 (FJS-12), etc.; and all are validated for use after QT reconstruction except FJS-12 [14, 15]. The FJS-12 was developed and has already been validated for assessing joint specific functional outcomes after arthroplasty [16]. In contrast to traditional instruments such as WOMAC, it has the advantage of being less influenced by a ceiling effect. However, it has not yet been validated for the assessment of functional outcomes after QT repair [17].

The main objective of this study was to validate the use of FJS-12 after QT repair surgery, in order to measure joint awareness. The primary hypothesis was that FJS-12 could be used for assessing knee specific functional outcomes after QTR surgical treatment.

## Methods

The present retrospective case series was conducted in a university-affiliated hospital and was approved by the Regional Ethics Committee of North-western and Central Switzerland (ID number 2021–01049). Patients who underwent surgical suture reconstruction following isolated traumatic QTR with the subsequent partial or total disruption of the extensor mechanism, between January, 2015 and January, 2020 were searched for in the hospital’s electronic data base. Out of the 84 initially found patients, 57 were included for the final analysis. Only patients who were of legal age and spoke German, French or English have been considered for enrolment. Additional exclusion criteria were represented by patient cognitive impairments, which made the completion of any questionnaire impossible or unreliable, and any later knee arthroplasties or distal femoral/proximal tibial osteotomies/fractures on the same knee. Patients were contacted by telephone and in writing to be included in the study. Patients who initially agreed to participate, but did not return the questionnaire after repeated reminders (at least three contact attempts) were also excluded from this study.

**Fig. 1:**
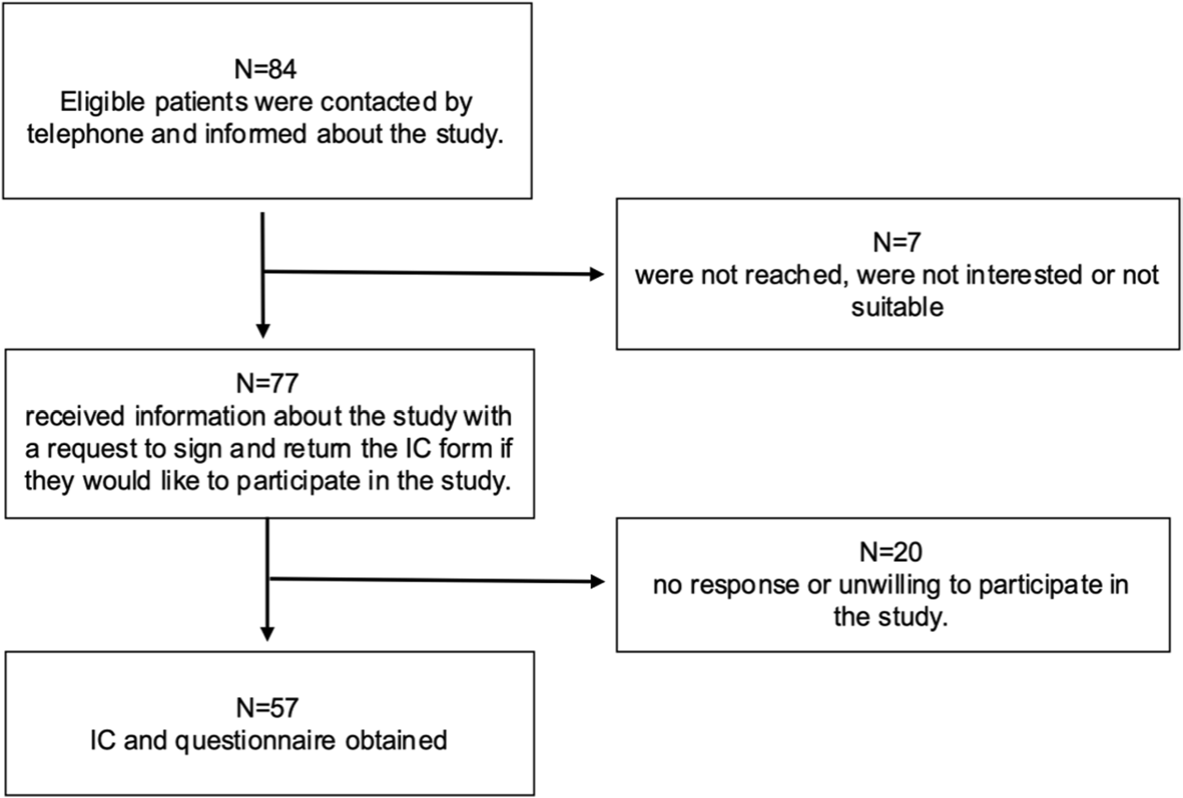
Flow chart of data collection

All methods were carried out in accordance with the Declaration of Helsinki. All participants were required to sign an informed consent prior to study inclusion. All the details regarding patients’ enrolment are illustrated in Fig. 1.

### Surgical technique

All patients were treated by transoseous suturing of the ruptured tendon. Under spinal anaesthesia, the patient was placed in the correct axial position in decubitus dorsalis. A longitudinal incision, from the superior patellar pole, approximately 10 cm proximal to the rupture area, was performed. Sharp and blunt dissection were required until the quadriceps tendon was exposed. In the next step, extensive lavage of the entire joint area, and careful debridement of the proximal patellar pole and the stump of the quadriceps tendon were performed. Fiberwire sutures were placed in the stump, and three transosseous tunnels were drilled in the proximal patella. Under traction, the Fiberwire sutures were crossed and passed through the drilled patellar tunnels, attaching the tendon to the patella. In order to check, the attachment to the proximal patellar pole, knotting the suture and palpating the quadriceps tendon was attempted. Joint capsule was closed using a 1-strand PDS suture with a transverse continuous suture. Extensive lavage and wound closure in layers, using subcutaneous and skin sutures were the final two steps. Postoperatively, the leg was immobilized in full extension and gradually flexed (30°/2 weeks) until 90°. Full weight bearing with the leg in extension was allowed.

### Outcome measures

General demographics like age, gender, body mass index (BMI), medications before surgery, activity at the time of injury, time interval from injury to surgery (in days) and were recorded as mentioned in Table1. All these information was checked in the medical records for authenticity. The primary outcome measure was FJS-12 which contains 12 questions and scores on Likert scale. For each question, the scoring method is from 0 to 4 (0, never; 1, almost never; 2, seldom; 3, sometimes; 4, mostly). Lower scores on the Likert scale shows less awareness of the operated knee during daily activities. The total score can be calculated by multiplying the mean value for the 12 included items by 25, and then subtract the obtained value from 100. The range of the final score is from 0 to 100 (where 0 is worst and 100 means best score) [18]. The WOMAC score, which was primarily developed to assess the outcomes of knee osteoarthritis, was also used in this study. However, this score has already been validated and is considered a reliable tool for ascertaining functional outcomes after QTR. It consists in 24 questions related to pain (5 questions), stiffness (2 questions) and daily life function [15]. The modified Lysholm knee score was also used in this study. It has been widely used and higher score indicates better knee function. Finally, TAS was also measured to evaluate the activity level of all patients. It represents a valid and reliable tool with universal acceptance [19].

**Table 1:** Study lot baseline characteristics

Patients characteristics	*N* = 57 *(mean* ± *SD)*
Age	69.2 ± 13.6
Gender	
*Male*	51 (89.5%)
*Female*	6 (10.5%)
Weight *(kgs)*	86.5 ± 17.1
Height *(cm)*	173.5 ± 7.6
BMI	28.6 ± 4.6
Previous injury to affected knee	
*Yes*	8 (14%)
*No*	49 (86%)
Tendon rupture	
*Complete*	42 (73.7%)
*Partial*	15 (26.3%)
Time interval from injury to surgery *(in days)*	3.3 ± 5.4
Comorbidities	
*Tumour*	1 (1.8%)
*Diseases of nervous system*	2 (3.5%)
*Respiratory diseases*	6 (10.5%)
*Cardiovascular diseases*	19 (33.3%)
*Gastrointestinal disorders*	8 (14%)
*Liver diseases*	1 (1.8%)
*Kidney disorders*	3 (5.3%)
*Metabolic disorders*	8 (14%)
*Skin diseases*	5 (8.8%)
*Musculoskeletal diseases*	9 (15.8%)
*Mental disorders*	2 (3.5%)
*Others*	3 (5.3%)
Operated dominant side	
*Yes*	39 (68.4%)
*No*	18 (31.6%)
Pre-operative TAS	4 ± 1

*SD* Standard deviation, *BMI* Body mass index, *TAS* Tegner activity scale

### Statistical analysis

A power analysis was performed. Approximately, 80 patients were thought to have had surgical repair of the quadriceps tendon in a span of 5 years (from January 2015 to January 2020) in the respective hospital. The response rate was assumed between 50 and 80%, therefore 50–80 patients were eligible. With 5% alpha error and 80% power, the effect size correlation was *r* = 0.38 and *r* = 0.31 for 50 and 80 patients respectively. All data was collected using RedCap, which is a secure electronic data capture (EDC) software (that allows patients to complete questionnaires online through an individual QR code), or in paper form, which was subsequently added to the electronic database by the study team. The statistical analysis was performed on IBM SPSS v 26.0 for Windows. Descriptive statistics were used to define all quantitative variables. The criterion and construct validity were assessed by Pearson correlation, which analysed FJS-12 and the other used PROMs (WOMAC, Lysholm and TAS). The principal component factor analysis was performed to investigate the internal consistency of FJS-12 along with Cronbach’s alpha value.

## Results

Fifty-seven patients returned the questionnaire and were enrolled in the study. All details regarding patients’ characteristics are presented in Table 1. The mean time from injury to surgery was 3.3 ± 5.4 days. Forty-two patients (73.7%) had a complete tendon rupture after injury and the dominant leg was more effected than the non-dominant one. Majority of our patients also had some associated comorbidities as mentioned in Table 1. However, all these were incidental findings. Some of the patients also reported the use of medications before surgery like corticosteroids (7%), statins (28%) and others (26%). Regarding the causes of rupture, 44 (77.2%) patients reported that they had indirect trauma such as falling, 3 (5.3%) patients had direct trauma (hitting with the object to the knee), 2 (3.5%), patients could not remember the cause and 8 (14%) of them had some other undefined causes of rupture. Activities at the time of injuries were also categorized to understand the extend of injury. Nine patients (15.7%) were walking while they injured their knee, running in 2 (3.5%) patients, escalating on an elevated surface in 25 (43.8%) patients, standing in 2 (3.5%) patients, jumping in 2 (3.5%) patients and other activity in 17 (30%) patients were reported as activities at the time of injury.

All patients reported satisfactory functional outcomes after surgery on FJS-12, WOMAC and Lysholm scores at mean follow-up time of 49.84 months ± 20.64 months, as shown in Table 2. However, the mean post- operative TAS decreased from level 4 to level 3 when compared to pre-operative score. To assess the construct validation, the FJS-12 was correlated with the other gathered PROMs (WOMAC, TAS, Lysholm) as presented in Table 2. There was a high negative correlation between WOMAC and FJS-12, but moderate positive correlations between FJS-12, Lysholm and TAS scores. The internal consistency reliability was calculated by using Cronbach’s alpha value, which was 0.96 for 12 items in FJS-12. With every variable left out, the Cronbach’s alpha would be slightly smaller or remain the same (i.e., “in bed at night” item) as mentioned in Table 3. It indicates that the factor structure of the FJS-12 is good and also suitable for measuring the knee outcomes after QTR.

**Table 2:** Patient reported outcomes

Post-operative scores	Follow-up time (mean ± SD)	Score value (mean ± SD)	Pearson coefficient (r) (FJS total score)
*Tegner score*	49.84 months ± 20.64 months	3 ± 1	0.25
*FJS total score*		59.3 ± 30.2	1
*WOMAC pain*		8.1 ± 4.1	-0.71
*WOMAC stiffness*		3.7 ± 1.8	-0.6
*WOMAC daily activities*		29.7 ± 14.7	-0.74
*Total WOMAC*		41.6 ± 19.8	-0.75
*Lysholm*		82.3 ± 16.9	0.66

*SD* Standard deviation, *FJS* Forgotten Joint Score, *WOMAC* Western Ontario and McMaster Universities Osteoarthritis Index

**Table 3:** Principal Component Analysis for all items of FJS-12

FJS-12 items	Scale Mean if Item Deleted	Scale Variance if Item Deleted	Corrected Item Total Correlation	Cronbach's Alpha if Item Deleted
*In bed at night*	**27.3**	**189,337**	**0.6**	**0.96**
*Sitting in a chair for more than an hour*	**26.8**	**182,964**	**0.7**	**0.95**
*Walk longer than 15 min*	**26.8**	**181,310**	**0.8**	**0.95**
*In the batht ub/shower*	**27.4**	**188,062**	**0.7**	**0.96**
*Drive*	**27.1**	**187,798**	**0.7**	**0.96**
*Climbing stairs*	**26.2**	**179,063**	**0.8**	**0.95**
*Walk on uneven ground*	**26.4**	**181,552**	**0.8**	**0.95**
*From a low-seated position (ex. couch)*	**26.3**	**180,638**	**0.7**	**0.96**
*Have to stand for a long time*	**26.8**	**181,002**	**0.8**	**0.95**
*do housework or gardening*	**26.3**	**175,229**	**0.8**	**0.95**
*Go for a walk or hike*	**26.4**	**175,216**	**0.8**	**0.95**
*Do sports*	**26.2**	**177,476**	**0.8**	**0.95**

*FJS* Forgotten Joint Score-12 Items

## Discussion

The main finding of this study was that the FJS-12 has shown a good reliability and adequate validity after QTR when compared to other widely used outcome measures. Our results regarding QTR outcomes are consistent with previous studies [20, 21].

FJS-12 was originally designed to assess the awareness of artificial joint after total knee arthroplasty [22]. Additionally, Behrend et al. [23] conducted a study to validate the FJS-12 for anterior cruciate ligament reconstruction (ACLR) as well, and they found that patients who underwent ACLR had lower FJS-12 score than non-operated individuals. Furthermore, in 2017, Behrend et al. also compared the FJS-12 scores with other outcome measures (WOMAC and KOOS) and they concluded that FJS-12 can be safely used for knee surgeries other than arthroplasty to evaluate the awareness of joint [24]. Recently, it has shown a good validity for osteotomies and meniscal surgeries [22–28]. Vermeijden et al. [25] reported a Cronbach’s alpha value of 0.89, which indicated reliable internal consistency and also reported acceptable construct validity (*r* = 0.62- 0.70) for primary ACLR. Lee JY et al. [26] reported Cronbach’s alpha of 0.9 with an inter-item correlation of 0.43 and a corrected total item of 0.68. Similarly, Kacmaz IE et al. [27] aimed to evaluate the joint awareness among patients who underwent isolated ACLR, ACLR + meniscectomy and ACLR + meniscal repair by using FJS-12. They have concluded that FJS-12 was highly correlated with the other commonly used scores. However, no previous study investigated the use of FJS-12 in patients who underwent QT repair after QTR. In the present study, the reported high consistency reliability (Cronbach’s alpha value of 0.9) of FJS-12 in assessing functional outcomes after QTR is consistent with the previously reported reliability values for ligament reconstructions and other knee related surgical procedures [26–28].

Conversely, Itoh et al. [28], which conducted a first study in order to validate the FJS-12 for medial opening wedge high tibial osteotomy, have reported a Cronbach’s alpha of 0.94 and correlation coefficient of 0.64–0.72 for all subscales of KOOS. This in turn indicated moderate to strong positive correlations between FJS-12 and KOOS. In our study, KOOS was not used, but negative correlations between FJS-12 and total WOMAC (*r* = -0.75) and moderate positive correlations with Lysholm and TAS were reported (*r* = 0.25 and *r* = 0.66), with a Cronbach’s alpha value for all 12 items in FJS-12 of 0.96 (Table 3). These similarities in the Cronbach’s alpha value for each included in FJS-12 item have not been previously reported in any study.

There are certain limitations of this study. Some of the patients affected by QTR were found to be older; therefore, no longer physically active. Used survey tools such as TAS or certain items of the FJS-12 (e.g. joint awareness during sport) could not address these patients, and contained lead to missing values in some patients. Another limitation is represented by the fact that the patients had to answer online or via a mailed questionnaire, and were not clinically assessed by a physician. Therefore, results were highly dependent on the subjective patient’s self-assessment. The accuracy of patients' answers can also be questioned due to the associated pain and discomfort that many of them reported as a result of pre-existing arthritic processes on the same knee. The small sample size and retrospective nature of this study make it difficult to estimate outcomes from different aspects. Future, prospective, experimental studies with larger sample size and long term follow ups are recommended in order to further explore and standardize the utilization of FJS-12 in a QTR setup.

## Conclusion

This study has found that FJS-12 is a reliable and easy to assess tool for functional outcomes after QTR surgery. It has shown moderate to strong correlation with other commonly used outcome measures (WOMAC, TAS and Lysholm).

## Data Availability

The datasets used and/or analyzed during the current study are available from the corresponding author on reasonable request.

## Abbreviations

ACLR: Anterior cruciate ligament reconstruction
BMI: Body mass index
EDC: Electronic data capture
FJS-12: Forgotten Joint Score- 12
KOOS: Knee Injury and Osteoarthritis Outcome Score
PROMs: Patient reported outcomes measures
QT: Quadriceps tendon
QTR: Quadriceps tendon ruptures
WOMAC: Western Ontario and McMaster Universities Osteoarthritis Index
